# Multisensory Stimulation in Rehabilitation of Dementia: A Systematic Review

**DOI:** 10.3390/biomedicines13010149

**Published:** 2025-01-09

**Authors:** Andrea Calderone, Angela Marra, Rosaria De Luca, Desirèe Latella, Francesco Corallo, Angelo Quartarone, Francesco Tomaiuolo, Rocco Salvatore Calabrò

**Affiliations:** 1 Department of Clinical and Experimental Medicine, University of Messina, Piazza Pugliatti, 98122 Messina, Italy; francesco.tomaiuolo@unime.it; 2 IRCCS Centro Neurolesi Bonino-Pulejo, S.S. 113 Via Palermo, C.da Casazza, 98124 Messina, Italy; angela.marra@irccsme.it (A.M.); rosaria.deluca@irccsme.it (R.D.L.); desiree.latella@irccsme.it (D.L.); francesco.corallo@irccsme.it (F.C.); angelo.quartarone@irccsme.it (A.Q.); roccos.calabro@irccsme.it (R.S.C.)

**Keywords:** multisensory stimulation, cognitive rehabilitation, dementia, Snoezelen therapies

## Abstract

**Background/Objectives**: Dementia leads to cognitive decline, affecting memory, reasoning, and daily activities, often requiring full-time care. Multisensory stimulation (MSS), combined with cognitive tasks, can slow this decline, improving mood, communication, and overall quality of life. This systematic review aims to explore methods that utilize MSS in the rehabilitation of patients with dementia. Its clinical value is rooted in its ability to offer a deep comprehension of how MSS can be successfully incorporated into rehabilitation treatments. **Methods**: Studies were identified from an online search of PubMed, EBSCOhost, Cochrane Library, Web of Science, Embase, and Scopus databases with a search time frame from 2014 to 2024. This review has been registered on Open OSF (n) 3KUQX. **Results**: Pilot studies investigating MSS interventions, encompassing Cognitive Stimulation Therapy (CST), Sonas therapy, and combined physical–cognitive exercise programs, have yielded mixed findings in individuals with dementia. CST has demonstrated significant improvements in general cognitive function, particularly in language skills, offering a promising approach for cognitive enhancement. Sonas therapy, while showing positive trends in some studies, does not consistently achieve statistically significant outcomes across all cognitive domains. Conversely, combined exercise programs have shown efficacy in improving dual-task performance, suggesting benefits for motor–cognitive integration. MSS delivered within specialized environments like Snoezelen rooms consistently produces positive effects on mood, reducing agitation and promoting relaxation. **Conclusions**: This review emphasizes how MSS can enhance cognitive, emotional, and behavioral results for individuals with dementia. It is essential for future research to standardize protocols, incorporate advanced technologies such as virtual reality, and rectify diversity gaps. Collaboration between different fields will improve the effectiveness and usefulness of MSS in caring for individuals with dementia.

## 1. Introduction

Dementia is a significant decrease in cognitive function that is severe enough to disrupt daily functioning, including memory, logic, communication, and daily tasks [1,2]. Over 55 million individuals around the globe are currently experiencing dementia, with nearly 10 million fresh instances documented each year. It commonly happens in elderly individuals but is not seen as a natural part of aging [3,4,5]. Symptoms consist of memory loss, difficulties with problem-solving and planning, confusion, changes in mood, and language impairment. The condition necessitates round-the-clock assistance and care in its later stages [6,7,8,9,10,11,12,13].

In the neurorehabilitation field, cognitive rehabilitation of dementia aims to maintain or improve existing cognitive skills by using strategies like mental stimulation therapy, reminiscence therapy, goal-oriented rehabilitation, and MSS approaches [14,15,16,17,18,19,20,21]. These methods typically focus on reducing cognitive decline and enhancing quality of life [22].

The dementia care approaches that involve MSS utilize the integration of different sensory inputs (touch, sight, sound, taste, and smell) during various activities, which helps stimulate both sensory and higher brain functions [23]. This method is based on the idea that dementia does not just impact memory and reasoning but also hinders sensory processing, reducing one’s ability to properly perceive and react to environmental stimuli [24,25].

Among the different types of sensory stimulation, the olfactory variety, which includes aromatherapy, has received considerable interest. The olfactory system is unique due to its direct connection to the limbic system and hippocampus, areas critical for memory and emotional processing. Aromatherapy involves the use of essential oils derived from plants, which emit potent scents that can evoke specific emotional responses or memories. Studies have demonstrated that exposure to pleasant and familiar smells can reduce agitation, alleviate anxiety, and improve cognition and mood in patients with dementia and mild cognitive impairment [26,27]. Aromatherapy, through olfactory stimulation, could also enhance sleep patterns and diminish behavioral issues like restlessness or aggression, commonly seen in the later stages of dementia [28,29]. Integrating olfactory MSS into everyday activities or treatment strategies offers a non-invasive and comprehensive method for dementia care, enhancing other cognitive and behavioral therapies. Furthermore, these interventions can trigger multiple areas of the brain at the same time, which can enhance neuroplasticity [30]. If these multisensory interventions in dementia are implemented in the early stages of the illness, neural pathways can be preserved before more severe cognitive decline and sensory impairments develop [31]. This has frequently resulted in better outcomes in terms of preserved cognitive abilities, communication skills, and emotional control [32]. Activities in multisensory engagement range from simple texture manipulation of objects like fabrics and natural materials to complex tasks [33]. One of the most extensively studied methods is Snoezelen therapy, where patients engage with controlled multisensory stimuli such as lights, sounds, and tactile sensations [34]. This treatment is especially advantageous for those with dementia, as it tackles sensory processing challenges and aids cognitive and emotional capabilities. During a Snoezelen session, participants are motivated to engage with these stimuli in manners that suit their comfort levels and capabilities. For individuals with dementia, this could mean either quietly appreciating the soothing environment or actively interacting with certain sensory aids under the supervision of a qualified guide [35]. Sessions generally include both passive involvements, like watching visual displays or hearing soothing sounds, and active engagement, such as feeling textured items or using sensory devices. The organized aspect of Snoezelen therapy distinguishes it from arbitrary sensory activities. It highlights the therapeutic application of particular stimuli to attain results like decreased agitation, better mood, improved communication, and reinforced caregiver–patient bonds. Research shows that Snoezelen therapy can foster moments of tranquility and connection, rendering it an important approach for individuals facing distress or behavioral issues in dementia [36,37].

This has effectively been proven to reduce irritation, enhance mood, and boost overall well-being in individuals with dementia [38]. Frequently, a cognitive task is paired with MSS to enhance memory and learning [39]. This can range from playing known songs and prompting the patient to recall memories to using visual aids in storytelling sessions. These actions involving the senses, along with cognitive tasks, enhance the immersion and effectiveness of the experience [40]. The reason for these sensory cognitive activities is that they provide chances for ongoing mental and emotional involvement [41]. Studies have demonstrated that extended exposure to multisensory environments can improve focus and working memory, as well as slow down the decline in language abilities. Engaged in mentally challenging tasks, sensory input would push the brain to process information in complex ways, aiding in maintaining cognitive flexibility and adaptability. The patient’s willingness to participate in this care is significantly increased through sensory experiences that evoke pleasant feelings or memories. Engaging in these activities is important for encouraging involvement in daily tasks such as getting dressed and eating, as well as socializing, which can become challenging with the presence of dementia [42].

This systematic review aims to explore methods that utilize MSS in the rehabilitation of patients with dementia. Its clinical value is rooted in its ability to offer a deep comprehension of how MSS can be successfully incorporated into rehabilitation treatments. This review fills an important gap in understanding how engaging multiple senses through MSS interventions can enhance cognitive, emotional, and functional outcomes in the dementia population by synthesizing existing evidence. Furthermore, recognizing the most impactful sensory combinations for various levels of cognitive impairment may assist practitioners in customizing interventions, thereby enhancing patient involvement and treatment results. With the rising global rates of dementia, the findings of this review may assist healthcare providers in adopting innovative, evidence-supported methods that foster a more comprehensive and individualized strategy for cognitive rehabilitation.

## 2. Materials and Methods

### 2.1. Search Strategy

**Figure 1:**
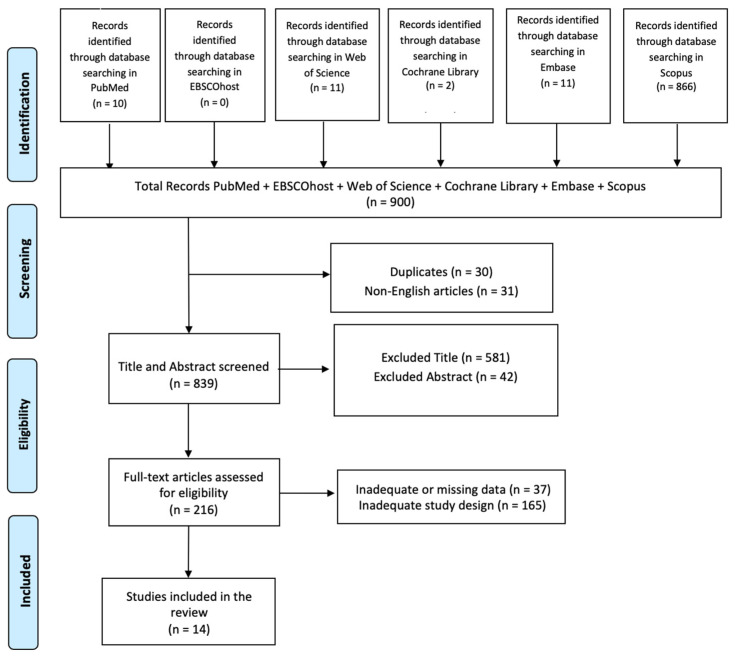
PRISMA 2020 flow diagram of evaluated studies.

This systematic review employs a structured method to assess research studies with a search time frame from 2014 to 2024. We conducted a comprehensive literature search (from 1 September 2024 to 20 December 2024) using PubMed, EBSCOhost, Web of Science, Cochrane Library, Embase, and Scopus databases, employing the keywords (All Fields: “Multisensory Stimulation”) AND (All Fields: “Cognitive rehabilitation”) AND (All Fields: “Dementia”). Searches were conducted independently by two reviewers (AC and DL) using Boolean operators and controlled vocabulary (e.g., MeSH terms) to enhance transparency and accuracy in identifying relevant studies. The PRISMA (Preferred Reporting Items for Systematic Reviews and Meta-Analyses) flow diagram was utilized to outline the process (identification, screening, eligibility, and inclusion) for selecting relevant studies as illustrated in Figure 1. The Cochrane Risk of Bias (RoB 2) tool was used to evaluate the risk of bias in randomized controlled studies, while the ROBINS-I tool was used for uncontrolled experimental papers in this review. Furthermore, all articles were screened based on titles, abstracts, and full texts by two researchers (AC and DL), who independently performed data extraction, article collection, and cross-validation to reduce the risk of bias (e.g., missing results bias, publication bias; time lag bias; and language bias). Collected data items encompassed study design, sample size, participant traits, types of dementia, details of the rehabilitation intervention, duration, outcomes assessed, and results. Data synthesis was performed utilizing both narrative approaches and quantitative analysis to address the diversity of study designs and types of dementia involved. Effect sizes, certainty of evidence, and statistical analyses from various study types representing different types of dementia were examined. This combined method facilitated a deeper grasp of the data, factoring in both qualitative insights and quantitative outcomes. These researchers (AC and DL) read full-text articles deemed eligible for this study, and in case of disagreement on inclusion and exclusion criteria, the final decision was made by a third researcher (RCS). Moreover, the agreement between the two reviewers (AC and DL) was assessed using the kappa statistic. The kappa score, with an accepted threshold for substantial agreement set at >0.61, was interpreted to reflect excellent concordance between the reviewers. This criterion ensures a robust evaluation of inter-rater reliability, emphasizing the achievement of a substantial level of agreement in the data extraction process. The list of articles was then refined for relevance, reviewed, and summarized, with key topics identified from the summary based on the inclusion/exclusion criteria. This review has been registered on Open OSF: DOI 10.17605/OSF.IO/3KUQX.

### 2.2. PICO Evaluation

We applied the PICO model (Population, Intervention, Comparison, and Outcome) to create our search terms. This systematic review analyzes individuals with dementia, including those at various stages of cognitive decline. The intervention will include methods using MSS in the process of rehabilitation. The comparison will assess the effectiveness of MSS-based interventions in comparison to standard care, other therapies not utilizing MSS, or no intervention. Improved cognitive function, increased happiness, higher life satisfaction, and reduced problematic behaviors and emotional challenges linked to dementia will be the primary outcomes.

### 2.3. Inclusion Criteria

The inclusion criteria for selecting studies in this systematic review aimed to identify research on MSS techniques employed in the rehabilitation of individuals experiencing dementia. Studies considered eligible concentrated on interventions that involved various senses (such as sight, sound, touch, taste, and smell) to evaluate their effects on cognitive, functional, or emotional results. Research featuring a definite explanation of the intervention and quantifiable or qualitative results concerning cognitive performance, overall well-being, or life quality were considered significant. Only studies released in English were considered to guarantee uniform interpretation and assessment. This review examined original research papers encompassing randomized controlled trials, non-randomized experimental studies, cohort studies, and longitudinal studies, as long as they described methodologies, intervention features, and outcome metrics associated with MSS in the rehabilitation of dementia.

### 2.4. Exclusion Criteria

The exclusion criteria were established to guarantee the inclusion of studies that closely matched the aims and scope of this systematic review. Reviews (including literature reviews, systematic reviews, integrative reviews, and narrative reviews) were not included, but their reference lists were analyzed for pertinent primary research. Moreover, research that employed animal models or focused on groups other than those with dementia was omitted to ensure consistency with the intended demographic of this review. Additional exclusions involved studies that concentrated exclusively on single sensory stimulation interventions (e.g., olfactory training or auditory stimulation) that did not incorporate multisensory methods, as this was inconsistent with the inclusion criteria. Studies exploring unrelated treatments or results that did not focus on cognitive, functional, or emotional rehabilitation in dementia were likewise excluded. Ultimately, conference abstracts, editorials, and opinion articles were excluded because of a lack of data for thorough assessment. A detailed summary of the systematic review methodology is visualized in Table 1.

## 3. Results

### 3.1. Quality of Included Studies—Risk of Bias

**Figure 2:**
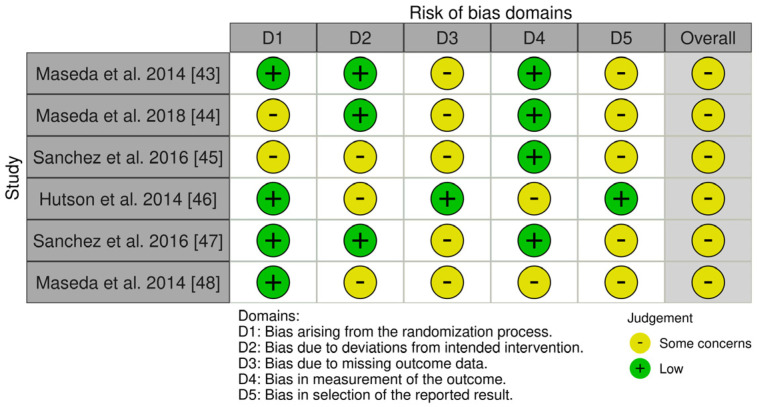
Risk of Bias (RoB) of included RCT studies.

We assessed the risk of bias using appropriate tools based on the design of the included studies [43,44,45,46,47,48,49,50,51,52,53,54,55,56]. Of the fourteen studies, six were a randomized controlled trial (RCT) [43,44,45,46,47,48]. For this one, we used the updated Cochrane Risk of Bias (RoB 2) tool, which covers five domains: (i) bias arising from the randomization process, (ii) bias due to deviations from the intended intervention, (iii) bias due to missing data on the results, (iv) bias in the measurement of the outcome, and (v) bias in the selection of the reported result (Figure 2) [57].

The evaluation of bias in the included studies highlighted strengths as well as areas of concern. Some studies showed strong methodologies in important areas, while others pointed out limitations that need to be addressed. In relation to the randomization process (D1), the majority of studies followed appropriate guidelines; however, issues were found in studies such as Maseda et al. [44] and Sanchez et al. [45], where unclear or inconsistent allocation methods may have affected the accuracy of the findings. In contrast, research by Maseda et al. [43], Hutson et al. [46], Sanchez et al. [47], and Maseda et al. [48] showed a low risk in this aspect, indicating a need for a more stringent randomization process. In most cases, there were very few instances of not following the planned interventions, as shown in Maseda et al. [43], indicating a high level of adherence to the protocol. Still, additional research from Sanchez et al. [45], Hutson et al. [46], and Maseda et al. [48] expressed doubts regarding the adherence to planned interventions (D2), which could impact the results. Improving the management of missing outcome data was identified as a necessary area for improvement. All studies included, except one, indicated issues with follow-up and completeness of reporting, raising concerns (D3). The lack of consistency in handling missing data could impact the overall trustworthiness of the results. The measurement of results (D4) was consistently strong in most studies. Maseda et al. [43] and Sanchez et al. [45] utilized trustworthy and accurate measures, which helped decrease the chance of measurement bias and bolstered trust in the documented results. Nevertheless, problems regarding the choice of disclosed outcomes were common. Research conducted by Maseda et al. [43,44] as well as Sanchez et al. [45] brought up concerns regarding transparency and potential signs of selective reporting (D5). This hinders trusting their conclusions completely because of lacking comprehensiveness and objectivity. In general, the studies that were included were classified as having “some concerns” regarding bias risk. Although overall acceptable, the methodological quality of this study could be improved in future research to address issues with randomization, data completeness, and selective reporting. Dealing with these obstacles would improve the dependability and relevance of discoveries in this area.

**Figure 3:**
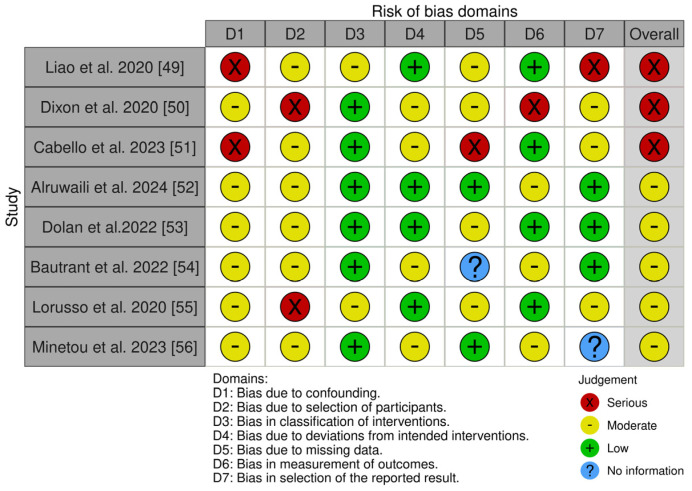
Cochrane Risk of Bias in Non-randomized Studies of Interventions (ROBINS-I).

For the eight non-randomized studies—three uncontrolled experimental studies [49,51,52], three qualitative studies [50,55,56], one prospective controlled study [53], and one retrospective cohort study [54]—we applied the ROBINS-I tool. ROBINS-I assesses bias in seven areas: (i) bias due to confounding, (ii) bias in participant selection, (iii) bias in classification of interventions, (iv) bias due to deviations from intended interventions, (v) bias due to missing data, (vi) bias in outcome measurement, and (vii) bias in selection of the reported outcome (see Figure 3) [58].

The examination of bias risk in the studies shows significant differences in quality, with many studies having weaknesses in certain aspects. An ongoing issue is the management of variables (D1) that may skew results, as demonstrated in studies by Liao et al. [49] and Cabello et al. [51] highlighting significant dangers. This suggests that external variables were not properly managed, which could compromise the credibility of their results. On the other hand, Alruwaili et al. [52] and Dolan et al. [53] demonstrated stronger performance compared to other studies, yet they still showed moderate risks in this area. The selection of participants (D2) was also identified as a crucial concern, with significant biases observed in research conducted by Dixon and Lorusso [50,55]. These results indicate difficulties in guaranteeing that selected individuals are a fair representation, potentially impacting the overall applicability of the findings. In most research projects, the categorization of treatments was effectively managed, and most showed minimal risks. Nevertheless, Lorusso et al. [55] and Alruwaili et al. [52] demonstrated some moderate risks, suggesting slight discrepancies or uncertainties in the definitions of interventions (D3). The studies showed that adherence to planned interventions was mostly at a low to moderate risk level, indicating that deviations did not greatly affect bias (D4). Nevertheless, the presence of missing data was a significant worry, especially in Cabello et al. [51], where critical problems were detected. This prompts concerns regarding the dependability of the findings and underscores the significance of strong data handling. Outcome assessment (D6) raised additional issues, with research by Dixon et al. [50] highlighting significant dangers. These results suggest there may be issues in how results were evaluated, leading to potential bias. In addition, most studies recognized selective reporting (D7) of results as a moderate risk, but Liao et al. [49] had a serious risk, suggesting possible concerns with transparency and completeness. In general, the research showed different levels of methodological rigor. These results highlight the significance of carefully evaluating the evidence, especially for research with significant biases. Additionally, it emphasizes the importance of future studies focusing on enhancing techniques to control confounding variables, improving participant selection, and handling incomplete data to increase the reliability and validity of the evidence base.

### 3.2. Synthesis of Evidence

In total, 900 articles were found: 30 articles were removed due to duplication after screening; 31 articles were excluded because it was not published in English; and 623 articles were excluded based on title and abstract screening. Finally, 202 articles were removed based on screening for inadequate and untraceable study designs (Figure 1). Fourteen research articles met the inclusion criteria and were therefore included in this review. These studies are summarized in Table 2.

This research explored the application of MSS in dementia treatment, incorporating sample sizes between 18 and 84 participants, mainly older adults (average age between 61.55 and 88.9 years, with some research targeting those aged 65 and above and others considering a broader spectrum from 68 to 102 years) diagnosed with dementia. Gender representation differed, as certain studies featured a predominance of female participants (as high as 95%), while others had both sexes represented (with percentages varying from 71.4% to 86.1% female) or did not indicate gender. The interventions included different MSS techniques, such as multisensory stimulation environments (MSSEs), Snoezelen therapy, individualized music therapy, aromatherapy, and technology-based methods utilizing devices like fiber-optic cables, water columns, vibrating water beds, tactile boards, video displays, mirror balls, interactive projection systems, and the ROXPro© system, along with environmental strategies like garden trips and the Sonas method (centered on music and poetry). The main senses engaged were visual, auditory, tactile, and olfactory, with a few studies also considering proprioceptive stimulation. The duration of treatment varied from one-time sessions to 16 weeks (with differing frequencies and lengths of sessions, like two 30 min sessions each week), and some studies featured follow-up periods. Outcome measures encompassed standardized tools such as the Mini-Mental State Examination (MMSE), Cohen–Mansfield Agitation Inventory (CMAI), Neuropsychiatric Inventory (NPI), Neuropsychiatric Inventory—Nursing Home (NPI-NH), Cornell Scale for Depression in Dementia (CSDD), Barthel index, Interact scale, biomedical metrics (heart rate and SpO2), Quality of Life–Alzheimer’s Disease (QoL-AD), Rivermead Behavioral Memory Test (RBANS), Delis–Kaplan Executive Function System (DKEFS), Token Test (TT), Behavioral Pathology in Alzheimer’s Disease Rating Scale (BANS-S), and the Holden Communication Scale, in addition to scales aimed at assessing behavioral, cognitive, and emotional states. Certain research utilized semi-structured surveys or qualitative interviews to collect information. Documented effect sizes ranged from small to large, with investigations often noting enhancements in agitation, mood, cognitive abilities, and social engagement. Particular results revealed notable decreases in agitation (CMAI scores), advancements in mood (greater happiness, social engagement, attentiveness, relaxation, and diminished boredom), along with improvements in cognitive function (MMSE scores, attention, language abilities, and short-term memory). Certain studies indicated that there were no substantial differences between the intervention and control groups, whereas others emphasized the distinct advantages of various MSS methods for specific symptoms (e.g., MSSE for wandering, Montessori-inspired therapy for agitation/aggression, and shouting). The statistical analyses conducted consisted of parametric techniques, including ANOVA and t-tests, non-parametric methods like the Mann–Whitney U and Kruskal–Wallis tests, as well as repeated measures ANOVA. Qualitative data analysis was conducted through thematic analysis and constructivist grounded theory. The certainty of evidence varied between low (in pilot or small-scale studies) and high (in studies with controlled designs and standardized measures), with certain studies clearly noting limitations stemming from small sample sizes or qualitative methods.

To further investigate all the obtained results, we sequentially summarized the primary insights regarding the use of MSS in the rehabilitation of people with dementia [43,44,45,46,47,48,49,50,51,52,53,54,55,56]. Three studies explored the effects of MSS on cognition [46,51,53]. Three papers examined the impact of MSS on mood and emotional well-being [44,48,49]. Other four research studies examined the effects on agitations and related behaviors [45,47,52,54]; meanwhile, the last four articles explored the role of environment and technology in MSS [43,50,55,56].

### 3.3. Effects of MSS on Cognition

Numerous pilot studies have studied the influence of MSS and associated therapies on the cognitive capacities of people with dementia, yielding varying results in terms of their effectiveness across different cognitive domains. A pilot single-blind prospective controlled trial (n = 28, mean age 80.29 years) compared CST and Sonas, a multisensory intervention involving the use of music, sounds, objects, and evocative activities to stimulate memory, emotions, and well-being, in individuals with moderate dementia. Within-group analysis revealed that CST significantly improved general cognitive function (MMSE, *p* = 0.032) and language skills (TT, *p* < 0.01, medium effect size 0.37). CST also showed significant improvement in verbal fluency compared to Sonas (*p* = 0.049), where scores slightly declined. No significant between-group differences were found for overall cognition or other cognitive measures like RBANS, memory, attention, or ADLs. While Sonas showed a non-significant trend towards improvement in MMSE scores, CST demonstrated clearer benefits for cognitive domains in this pilot study [53]. An uncontrolled experimental study (n = 19, dementia diagnosis) evaluated a home-based, mobile health technology-delivered exercise program combining cognitive, multisensory, and physical elements on dual-task performance. Participants were assigned to an experimental group (EG, n = 12) receiving 24 training sessions or a control group (CG, n = 7) receiving standard care. The results showed the EG significantly improved their ability to walk while performing a verbal fluency task (naming animals) post-intervention, unlike the CG, whose performance worsened. No significant changes were observed in single-task gait, strength, or other cognitive measures for either group. This suggests that mobile health-guided, combined exercise can positively impact dual-tasking in dementia, warranting further investigation with larger trials [51]. A pilot randomized controlled trial (n = 39, moderate to severe dementia) assessed the feasibility and effectiveness of Sonas, a group intervention combining MSS, reminiscence, and light physical activity. Participants were randomized to receive 14 Sonas sessions or treatment as usual. Despite good attendance at Sonas sessions (mean 12.4/14), no statistically significant differences were found between groups in cognition, quality of life, communication, depression, anxiety, or behavioral disturbances. This pilot study suggests Sonas did not demonstrate significant benefits in these outcomes, although it was well tolerated. Further research with larger samples is needed to explore Sonas’s potential [46]. In essence, these pilot studies suggest that MSS can have positive effects on certain cognitive functions in dementia but that its efficacy is not universal and requires careful consideration of the specific intervention, the targeted cognitive domains, and rigorous research methodologies.

### 3.4. Impact of MSS on Mood and Emotional Well-Being

A collection of studies investigating the effects of various MSS approaches on individuals with dementia, including Snoezelen room environments, individualized music, and natural outdoor settings, reveals consistent positive effects on mood, behavior, and related physiological measures, with variations depending on the intervention and context. A randomized controlled trial (n = 21, severe dementia, mean age 88.9 years) compared the effects of MSSE in a Snoezelen room and individualized music sessions on mood, behavior, and biomedical parameters. Both interventions, delivered in two weekly 30 min sessions for 12 weeks, yielded immediate positive effects on mood and behavior, with significant improvements in happiness/contentment, spontaneous speech, social interaction, attentiveness, enjoyment, reduced boredom, and relaxation. While the MSSE group showed better visual tracking of stimuli during sessions, the music group exhibited greater relaxation during sessions. Both groups experienced a decrease in heart rate and an increase in oxygen saturation post-intervention, with no significant between-group differences. These findings suggest both MSSE and individualized music are effective non-pharmacological approaches for managing mood and behavioral disturbances in severe dementia [44]. Maseda et al. [48] (n = 30, dementia diagnosis) compared MSSE in a Snoezelen room with one-to-one activity sessions over 16 weeks, assessing mood, behavior, and biomedical parameters. Both interventions led to immediate improvements in mood and behavior post-session, including increased spontaneous speech, better social interaction, greater attentiveness, increased activity/alertness, reduced boredom, and enhanced relaxation/contentment. Both groups also showed decreases in heart rate and an increase in oxygen saturation after sessions. No significant differences were found between MSSE and activity sessions for any measured outcomes. These findings suggest both MSSE and one-to-one activities offer similar short-term benefits for mood, behavior, and physiological states in individuals with dementia [48]. Liao and colleagues [49] (n = 42 staff members from nine dementia care facilities) explored the perceived benefits of garden visits for residents with dementia. Staff reported that garden visits positively influenced mood, social interaction, depression, and agitation, attributing these effects to the natural environment’s MSS. Improvements in attention and time orientation were also noted. Notably, staff from facilities with free garden access reported significantly greater positive effects on mood, long-term memory, language, spatial ability, aggression, and agitation compared to staff from facilities with restricted access. These findings suggest that garden access, particularly unrestricted access, may offer therapeutic benefits for individuals with dementia, impacting both mood and certain cognitive functions [49]. The studies provide compelling evidence for the positive impact of MSS on mood and emotional well-being in dementia. However, they also emphasize that the specific benefits can vary depending on the type of MSS, the context in which it is delivered, and the individual’s needs.

### 3.5. Effects of MSS on Agitation and Related Behaviors

This paragraph includes publications on the influence of MSS on agitation and related behaviors in dementia patients, which provide encouraging evidence of efficacy, with outcomes changing depending on the individual MSS approach, target behaviors, and cultural setting. A randomized controlled trial (n = 32, severe dementia) compared MSSE with one-to-one activity sessions and a control group over 16 weeks, assessing agitation, mood, cognition, and dementia severity. While both MSSE and activity interventions reduced aggressive behavior and overall agitation during the intervention period, with diminishing effects at follow-up, MSSE demonstrated greater improvements in neuropsychiatric symptoms and dementia severity compared to both activity and control groups. However, no significant differences were found between groups regarding mood or cognitive function. These findings suggest that MSSE may offer specific benefits for managing agitation and dementia severity beyond those of standard one-to-one activities [45]. Alruwaili et al. [52] investigated a culturally adapted multisensory intervention for managing agitation in Arab elders with dementia. Thirty-one patients received the intervention, which combined Snoezelen (controlled sensory stimulation), aromatherapy, and personal items from the patients’ backgrounds, while thirty-one patients served as controls. The intervention group showed significant reductions in agitation behaviors and improvements in quality of life compared to controls. These findings suggest that this culturally sensitive approach may be a valuable tool for reducing agitation and improving well-being in Arab dementia patients [52]. A retrospective study (n = 84, institutionalized older adults, primarily with dementia) investigated the efficacy of tailored non-pharmacological therapies (NPTs) for nocturnal behavioral and psychological symptoms of dementia (BPSDs). Wandering, agitation/aggression, and screaming were the most prevalent BPSDs. MSS proved most effective for wandering, while Montessori-based therapy was most effective for agitation/aggression and screaming, showing significant differences compared to other NPTs like cognitive stimulation and reminiscence. These findings support the use of tailored NPTs by trained staff for managing specific nocturnal BPSDs in long-term care settings [54]. Another randomized controlled trial (n = 22 at baseline, n = 18 at follow-up, severe/very severe dementia) compared MSSE with personalized music sessions over 16 weeks, assessing agitation, mood, anxiety, cognition, and dementia severity. While both interventions reduced overall agitation during the session, MSSE produced a significantly greater improvement in anxiety and dementia severity than personalized music. Although mood declined in the music group during the intervention but recovered in both groups at follow-up, and cognition declined similarly in both groups, these differences were not statistically significant. These findings suggest that MSSE may provide greater benefits for anxiety and dementia severity in severe dementia than personalized music [47]. The data suggest that MSS is a promising non-pharmacological treatment for dementia-related agitation and behaviors. Maximizing the success of the MSS technique necessitates adapting it to the individual’s needs and cultural environment, as well as considering the specific target behaviors.

### 3.6. Role of Environment and Technology in MSS

**Figure 4:**
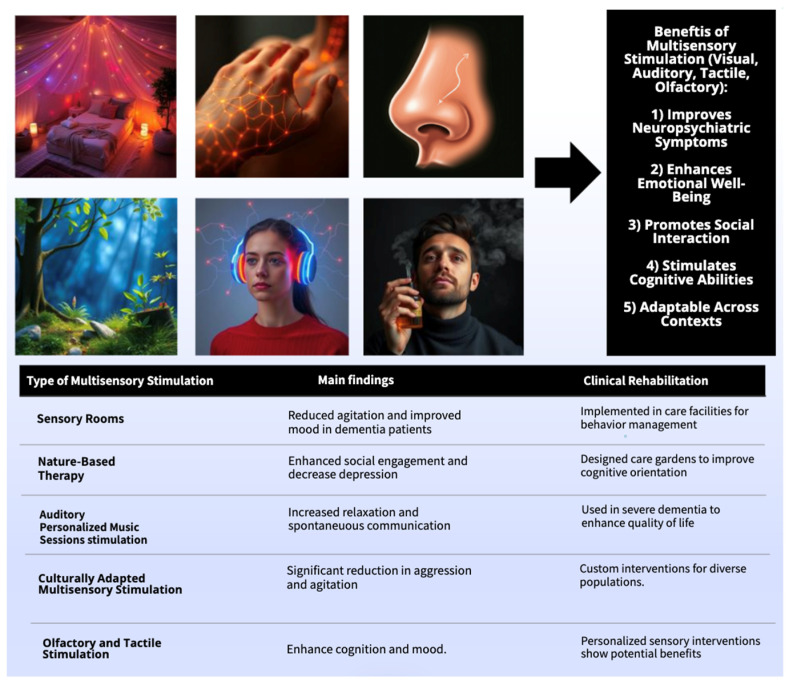
Key findings of the studies.

This last paragraph explores the role of environment and technology in MSS for individuals with dementia and reveals the potential benefits of MSS interventions while also highlighting key implementation challenges and the importance of personalized approaches and technological adaptations. A randomized controlled study (n = 30, dementia) compared an MSSE to individualized activities and a control group over 16 weeks, assessing behavior, mood, cognition, and functional impairment. While both MSSE and activity groups showed behavioral improvements and higher agitation scores (CMAI, NPI-NH) during the intervention, with no significant differences between them, MSSE showed a significant improvement in physically non-aggressive behaviors compared to the activity group. However, these positive effects, along with improvements in mood, did not persist at the 8-week follow-up [43]. A qualitative study explored the implementation and perceived effectiveness of multisensory environments (MSEs) in Veterans Health Administration (VHA) community living centers for veterans with dementia. Interviews were conducted with 32 staff members from 12 of the 53 VHA sites. Staff perceived MSEs as effective in improving behavior, particularly in reducing agitation and promoting calming effects, with music, aromatherapy, and bubble tubes being favored elements. Key barriers to MSE uptake included insufficient staff training (especially regarding therapeutic application and maintenance), lack of dedicated MSE rooms or adequate space, and staff turnover. These findings suggest that while MSEs are a promising intervention, addressing implementation barriers through improved training, dedicated spaces, and staff engagement is crucial for optimizing their effectiveness [55]. Another qualitative study (n = 50; 30 people with dementia, 10 nurses/caregivers) explored the design and development of interactive spatial objects with embedded technology to enhance the well-being of individuals with dementia in long-term care settings. The prototypes included interactive armchair simulating seaside experiences, a wall frame with visual and auditory elements triggered by proximity sensors, and a handrail cover activating a narrated story. These prototypes were designed to stimulate engagement, facilitate communication, and support a comforting narrative environment. Evaluation of their impact on residents’ well-being is ongoing through sensory ethnography methods [56]. An uncontrolled experimental study explored the intersection of technology use and sensory changes in individuals with mild to moderate dementia. Through interviews with 11 individuals with dementia (average age 61.55, predominantly Caucasian) and 19 practitioners (all female, predominantly Caucasian), the study identified three key strategies for accommodating sensory changes: stimulating at a desired level (e.g., using noise-canceling headphones), adjusting built-in technology settings (e.g., font size and voice assistants), and switching devices (e.g., from smartphones to tablets). When these strategies proved inadequate, participants ceased using certain technologies, particularly those causing sensory overload like cluttered websites or complex social media platforms. The study highlights the need for technology design to consider intentional sensory stimulation, tailoring input to individual needs, and avoiding overstimulation [50]. These studies demonstrate that environment and technology play crucial roles in MSS interventions for dementia. While MSS shows potential for improving behavior and well-being, successful implementation requires careful consideration of practical barriers, personalized approaches, and adaptable technology design that addresses the unique and changing sensory needs of individuals with dementia. A summary of the key findings of the results section is displayed in Figure 4.

## 4. Discussion

The research reviewed in this article offers important knowledge on how MSS interventions can effectively help in the rehabilitation of dementia patients, especially in institutional environments. While a consistent theme across the literature suggests the potential benefits of MSS, the specific outcomes and the magnitude of effects vary considerably depending on the intervention type, target population, and methodological rigor of the study. For instance, some interventions, such as CST, have demonstrated significant improvements in specific cognitive domains like language and general cognitive function, particularly within-group analyses. Conversely, other approaches, like Sonas, have yielded less consistent results in cognitive outcomes, despite showing potential for positive effects on mood and well-being. Furthermore, studies employing combined interventions, such as mobile health-guided dual-task training, have highlighted the potential for enhancing specific cognitive abilities like verbal fluency during ambulation. These discrepancies underscore the need for careful consideration of intervention design, targeted outcomes, and rigorous research methodologies to fully understand the potential of MSS interventions in dementia care. This discussion will delve into these specific findings, comparing and contrasting the results across studies to provide a more nuanced understanding of MSS efficacy.

### 4.1. Clinical Applications of MSS in Cognitive Rehabilitation of Dementia

The reviewed studies offer valuable insights into the potential clinical applications of MSS for cognitive rehabilitation in dementia while also highlighting the need for careful consideration of intervention design and targeted outcomes. A key finding is the differential effectiveness of various MSS approaches. For instance, the pilot study comparing CST and Sonas demonstrated that CST yielded significant improvements in general cognitive function (MMSE) and language skills (TT), with a medium effect size for the latter, and also outperformed Sonas in verbal fluency. Conversely, Sonas intervention, while well tolerated, did not show statistically significant benefits on cognitive measures. This discrepancy underscores the importance of intervention specificity: Structured, targeted programs like CST, which focus on specific cognitive domains through group activities and discussions, seem to offer more tangible benefits than more general MSS approaches like Sonas. This is further supported by the study of Cabello et al. [51] investigating a home-based, mobile health-delivered exercise program combining cognitive, multisensory, and physical elements. This intervention demonstrated significant improvements in dual-task performance, specifically the ability to walk while performing a verbal fluency task (naming animals). This finding suggests that integrating MSS with physical activity and technology can be a promising avenue for enhancing functional cognition [59,60]. In practical terms, these findings suggest that clinicians could prioritize structured cognitive approaches like CST when dealing with specific cognitive deficits in dementia patients. Furthermore, the successful integration of MSS with mobile health technology and exercise opens up new possibilities for delivering personalized, home-based rehabilitation programs that address both cognitive and physical decline [51]. However, given the limited sample sizes and varying methodologies across these pilot studies, larger, randomized controlled trials are crucial to confirm these findings and establish clear clinical guidelines for the optimal application of MSS in dementia care. These trials should focus on specific cognitive domains, using standardized assessments, and comparing different MSS approaches to determine which interventions are most effective for different patient profiles and stages of dementia. Beyond the modalities explored in the primary studies discussed, emerging evidence suggests that olfactory stimulation also holds promise as a valuable tool in cognitive rehabilitation for dementia [61,62,63,64,65]. Studies by Cha et al. (2022) [26] and Chen et al. (2022) [27] have specifically investigated the impact of olfactory training on cognitive function. Cha et al. (2022) explored the effects of intensive olfactory training on patients with dementia [26], while Chen et al. (2022) focused on the impact of olfactory training on olfaction, cognition, and brain function in patients with mild cognitive impairment [27]. These studies indicate that targeted olfactory stimulation can positively influence cognitive performance, suggesting that integrating olfactory training into multisensory interventions could further enhance their therapeutic potential [26,27]. This reinforces the need for a comprehensive and multifaceted approach to MSS, incorporating various sensory modalities to maximize cognitive benefits for individuals with dementia.

### 4.2. Clinical Applications of MSS for Mood and Behavioral Management in Dementia

MSS offers promising avenues for enhancing mood and emotional well-being in individuals with dementia, though the effectiveness of specific interventions depends on several factors. Notably, both Snoezelen environments and individualized music have demonstrated consistent positive effects. Maseda et al. [44] observed immediate improvements in various aspects of emotional well-being in individuals with severe dementia following both Snoezelen sessions and individualized music interventions. These improvements encompassed positive affect (happiness/contentment), spontaneous speech, social interaction, attentiveness, enjoyment, reduced boredom, and relaxation. While Snoezelen therapy appeared to enhance visual tracking, individualized music seemed particularly effective in promoting relaxation. This suggests that these distinct MSS approaches may engage different sensory modalities and consequently influence different facets of emotional experience. Physiological changes, such as a decreased heart rate and increased oxygen saturation, further corroborated the positive impact of both interventions on relaxation and physiological arousal. These short-term benefits were mirrored in the longer-term study by Maseda et al. [48], who compared Snoezelen with one-to-one activity sessions over 16 weeks in individuals with a dementia diagnosis. While both interventions yielded similar improvements in mood, behavior, and physiological states, no significant differences were found between them, suggesting that both structured activities and enriched sensory environments can contribute to emotional well-being. Furthermore, the importance of environmental context is highlighted by Liao et al. [49], whose research demonstrated the positive influence of garden visits on mood, social interaction, depression, and agitation in residents of dementia care facilities. Importantly, unrestricted access to gardens was associated with more pronounced benefits across a wider range of outcomes, including long-term memory, language, spatial ability, and even reduced aggression, emphasizing the therapeutic value of natural environments as a form of MSS. Clinically, these findings advocate for a multifaceted approach to MSS, integrating structured interventions (such as individualized music and one-to-one activities) with access to enriching environments like Snoezelen rooms and gardens. Tailoring interventions to individual preferences and needs is crucial, recognizing that some individuals may respond more favorably to stimulating environments like Snoezelen, while others may benefit more from calming interventions like music [66,67,68,69,70]. Prioritizing access to natural environments, particularly garden access, can be a key consideration in dementia care settings. Future research should investigate optimal combinations of MSS interventions for different dementia subtypes and specific behavioral and emotional symptoms.

### 4.3. MSS and Neuroplasticity in Dementia Rehabilitation

The research on MSS in dementia suggests a promising link between these interventions and neuroplasticity, the brain’s capacity to reorganize itself by forming new neural connections. Although dementia progressively impairs this capacity, evidence indicates that residual plasticity can be leveraged to mitigate cognitive and functional decline [71,72,73]. MSS, by simultaneously engaging multiple sensory modalities, can create a rich and stimulating environment that promotes neural activity and connectivity. This multisensory input may trigger several neuroplastic mechanisms. Firstly, it can strengthen existing neural pathways by increasing synaptic transmission and efficiency. Repeated activation of specific neural circuits through targeted sensory stimulation can enhance their functionality and resilience against further neurodegeneration [74]. Secondly, MSS can facilitate the formation of new neural connections, or neurogenesis, particularly in brain regions associated with learning, memory, and sensory processing. By providing diverse sensory experiences, MSS may stimulate the release of neurotrophic factors, such as brain-derived neurotrophic factor (BDNF), which support neuronal survival, growth, and differentiation [75]. Thirdly, MSS can enhance functional connectivity between different brain regions. By integrating information from various sensory modalities, the brain is forced to coordinate activity across multiple neural networks, strengthening their connections and improving overall brain efficiency. This enhanced connectivity can be particularly beneficial for complex cognitive functions that rely on the interplay of multiple brain areas. Furthermore, the positive emotional and behavioral effects of MSS, such as reduced agitation and improved mood, can indirectly support neuroplasticity. By reducing stress and creating a more positive emotional state, MSS can create a more conducive environment for learning and neural reorganization. In practical terms, this suggests that implementing well-designed MSS programs can potentially slow down the progression of cognitive and functional decline in dementia by maximizing residual neuroplasticity. By providing personalized and engaging sensory experiences, clinicians can create opportunities for the brain to reorganize and adapt, leading to more effective rehabilitation outcomes and improved quality of life for individuals with dementia. Further research is needed to fully understand the specific mechanisms by which MSS influences neuroplasticity in dementia and to determine the optimal parameters for maximizing its therapeutic benefits. Table 3 showcases the benefits and descriptions of different MSS related to dementia conditions.

### 4.4. Strengths and Limitations

This systematic review points out various strengths and limitations that offer valuable insight into research on MSS in dementia rehabilitation. One significant advantage is the thorough and organized approach taken. By incorporating various databases, such as PubMed, EBSCOhost, Web of Science, Cochrane Library, Embase, and Scopus, a wide array of studies was taken into account, while following PRISMA guidelines and utilizing tools like Cochrane RoB 2 and ROBINS-I increased the transparency and robustness of the selection process. Moreover, employing the kappa statistic for assessing agreement between reviewers guaranteed a high level of reliability in data extraction. The addition of both storytelling and numerical combination enhances the review even more, enabling a fair evaluation of subjective perspectives and numerical results. Another important advantage is the use of the PICO model, which offers a precise structure for identifying the population, intervention, comparison, and outcomes. This method allowed for a concentrated study on the impact of MSS-based treatments on cognitive, emotional, and functional results in individuals with dementia. Additionally, clearly defining the criteria for inclusion and exclusion ensured consistency and relevance.

Nevertheless, there are limitations to the review. A major obstacle is the likelihood of publication bias due to the inclusion of only English language studies. This could lead to the exclusion of important studies in different languages, which might impact the completeness of the results. Although an extensive search strategy was used on significant databases, this study only included research from 2014 to 2024, possibly missing out on important studies before that period. Numerous studies showed moderate-to-high levels of bias, especially in relation to randomization, adherence to intervention protocols, and handling of missing data. Another constraint is the dependence on studies of differing quality, some of which displayed shortcomings in controlling confounding factors and ensuring the selection of representative participants. These problems could affect how applicable the results are in a broader context. Moreover, the decision to exclude animal studies and non-dementia populations, although needed for specificity, could restrict the investigation of wider implications of MSS. Using bibliographies from different systematic reviews to find studies is another constraint, as it could affect the repeatability, which is crucial and may also lead to bias. While ideally, only studies with homogenous designs would be included for quantitative synthesis, the limited number of studies specifically investigating MSS in dementia necessitated the inclusion of various study designs (including randomized controlled trials, non-randomized trials, and uncontrolled studies) to provide a comprehensive overview of the available evidence. This heterogeneity, however, limits the ability to perform robust statistical comparisons and direct comparisons of efficacy between different MSS approaches. This limitation is acknowledged, and the findings should be interpreted with caution, considering the potential for bias and confounding introduced by the different methodological approaches. More research is needed on the long-term effects of MSS as existing studies have mainly looked at immediate, short-term results.

## 5. Conclusions

This systematic review emphasizes the benefits of MSS in enhancing cognitive function, emotional well-being, and behavioral outcomes in dementia patients. Even though MSS-based interventions are showing potential in reducing neuropsychiatric symptoms and improving quality of life, inconsistency in methods and results among studies highlights the importance of standardization in upcoming research. Future prospects should prioritize longitudinal research to evaluate lasting impacts, improvement of MSS protocols customized for personal requirements, and incorporation of cutting-edge technologies such as virtual reality to improve sensory involvement. Additionally, it will be essential to address gaps in participant diversity and expand research in community settings in order to generalize findings. Enhanced collaboration among various fields, such as psychology, neurology, and rehabilitation sciences, can enhance the evidence base and enhance the practical use of MSS interventions for dementia care.

## Figures and Tables

**Table 1:** Detailed summary of the systematic review methodology.

Section Methodology	Details
Search Strategy	Databases: PubMed, EBSCOhost, Web of Science, Cochrane Library, Embase, and Scopus. Keywords/Search String: (All Fields: “Multisensory Stimulation”) AND (All Fields: “Cognitive Rehabilitation”) AND (All Fields: “Dementia”). Boolean operators and controlled vocabulary (e.g., MeSH terms) were used.
Search Period	Search Time Range: studies published between 2014 and 2024. Time Search Conduction: from 1 September to 20 December 2024.
Study Selection	Two reviewers (AC and DL) independently screened articles at title, abstract, and full-text levels. Disagreements resolved through discussion or by a third reviewer (RSC). PRISMA flowchart used to visualize selection process.
Tool Used	Risk of Bias Tools: RoB 2 for randomized controlled trials and ROBINS-I for non-randomized studies. Inter-Rater Agreement: Kappa statistic used to measure inter-rater reliability (threshold for substantial agreement: kappa > 0.61).
Inclusion Criteria	Primary research focused on multisensory stimulation in rehabilitation for dementia. Studies must include well-defined interventions using multiple senses and report measurable outcomes (e.g., cognitive function, well-being, and life quality). Published in English.
Exclusion Criteria	Excluded reviews, animal studies, and studies focusing on single-sensory stimulation (e.g., olfactory training). Conference abstracts, editorials, and opinion articles were also excluded due to insufficient data for assessment.
Data Extraction	Extracted data included study design, sample size, participant characteristics, intervention details (e.g., sensory types, duration, etc.), outcomes assessed (e.g., cognitive performance, emotional well-being, etc.), and findings.
Synthesis Approach	Narrative synthesis combined with quantitative analysis. Effect sizes, evidence certainty, and statistical outcomes analyzed to address varied study designs and dementia stages.
PICO Evaluation	Population: individuals with dementia undergoing cognitive rehabilitation. Intervention: multisensory stimulation techniques. Comparison: standard care, other therapies, or no intervention. Outcome: improved cognitive function, emotional well-being, and quality of life.
Articles Included per Database	PubMed: 11; EBSCOhost: 0; Web of Science: 0; Cochrane Library: 0; Embase: 0; Scopus: 3; Total articles included: 14

Legend: Preferred Reporting Items for Systematic Reviews and Meta-Analyses (PRISMA), Cochrane Risk of Bias 2 (RoB 2), Risk of Bias in Non-randomized Studies of Interventions (ROBINS-I).

**Table 2:** Summary of studies included in the research.

Author	Aim	Study Design/Intervention	Treatment Period	Sample Size/Sample Characteristics	Outcomes Measures	Type of Multisensory Stimulation/Senses Involved/Main Findings	Effect Size/Certainty of Evidence	Statistical Analysis/Magnitude of Effect
Maseda et al. 2014 [43]	To explore how MSSE, activity sessions, and a control group impact the behavior, mood, cognitive function, and ADLs in dementia patients.	Randomized Controlled Trial.	The program lasted 16 weeks, with each group having two 30 min sessions per week, and was followed by an 8-week follow-up period.	Size: 30 participants. Age: mean (SD): 87.3 (5.3) years; minimum–maximum: 77–96 years. Gender: female: 27 (90%); male: 3 (10%).	CMAI, NPI-NH, CSDD, and MMSE.	Multisensory stimulation: employed fiber-optic cables, water columns, vibrating water beds, aromatherapy, tactile boards, music, video displays, and mirror balls, among various others. Senses involved: visual, auditory, tactile, and olfactory senses. Main findings: MSSE sessions led to more significant decreases in agitation (CMAI scores) than both activity-based sessions and control groups. Enhancements were observed in emotional and functional areas (Barthel index). Behavioral symptoms (NPI-NH scores) demonstrated considerable improvement. Cognitive results (MMSE scores) showed slight improvement, but they were not the main emphasis.	Effect size: large effect: verbally agitated behavior (F = 10.540, *p* < 0.001). Medium effect: physically nonaggressive behavior (F = 4.172, *p* = 0.023), NPI-NH total behavior (F = 4.513, *p* = 0.018), and mood (CSDD scores, F = 6.166, *p* = 0.024). Small-to-medium effect: occupational disruptiveness (F = 3.575, *p* = 0.040), cognitive level (GDS scores, F = 5.457, *p* = 0.038), and functional status in ADL (Barthel index). Certainty of evidence: high for the managed design and standardized measures applied, although the sample size restricts generalizability.	Student t-tests for continuous data. Chi-square tests for nominal variables. Data were summarized using frequency, percentage, mean, and standard deviation. Notable disparities in agitation reduction, behavioral symptoms, and functional enhancements between the MSSE group and others indicate a significant therapeutic effect.
Maseda et al. 2018 [44]	To examine the impacts of MSSE and personalized music therapy on mood, behavior, and biomedical measurements in elderly individuals with dementia.	Randomized Controlled Trial.	12 weeks consisting of 24 sessions, each with a duration of 30 min.	Size: 21 participants. Age: The mean age of participants was 88.9 years (SD ± 6.69). Gender: 71.4% females.	Interact scale and biomedical parameters.	Multisensory stimulation: Snoezelen therapy. Senses involved: visual, auditory, tactile, and olfactory stimulation. Main findings: Both groups exhibited notable enhancements in mood, such as heightened happiness/contentment, increased social interaction, and better attentiveness. The MSSE group demonstrated more pronounced improvements in relaxation and alertness in comparison to the individualized music group.	Effect size: Significant improvements were noted in mood and behavior, including increased happiness/contentment (*p* = 0.001). Better social engagement (*p* = 0.023). Improved attentiveness/focus (*p* = 0.005). Enhanced alertness or activity (*p* = 0.017). Reduced boredom (*p* = 0.026). Increased relaxation/contentment or appropriate sleeping (*p* = 0.021). Individualized music group: Participants showed improvements in increased happiness/contentment (*p* = 0.013). Better social engagement (*p* = 0.034). Improved attentiveness/focus (*p* = 0.007). Certainty of evidence: moderate, based on the noted statistical significance in mood and behavior enhancements without variance between groups.	Paired t-tests were employed to evaluate changes in mood and behavior within the group (e.g., prior to and following the intervention). A two-way mixed ANOVA with repeated measures examined group differences in changes in mood and behavior. Indicators of effect size: Eta-squared (η^2^) was utilized, with small, medium, and large effects reflecting values of 0.02, 0.13, and 0.26, accordingly. The notable results indicated substantial impacts on mood and behavior enhancements (η^2^ > 0.26).
Sanchez et al. 2016 [45]	To assess the impact of an MSSE in addressing agitation, mood, and cognitive function among elderly individuals with severe or very severe dementia, as compared to activity-based therapy and a control group.	Randomized Controlled Trial.	16 weeks.	Size: 32 participants. Age: 68–102 years range. Gender: females 25 (78.1%) and males 7 (21.9%)	CMAI, CSDD, SMMSE, BANS-S, and NPI.	Multisensory stimulation: fiber-optic cables, water bubble columns, a water bed, a rotating mirror ball, video, interactive projecting system, music, and aromatherapy with fragrant oils. Senses involved: visual, auditory, tactile, and olfactory stimulation. Main findings: Members of the MSSE group exhibited notable enhancements in behavior, mood, and cognitive status relative to both the control group and the activity group, especially concerning agitation, depressive symptoms, and cognitive performance (assessed via the SMMSE and NPI).	Effect size: Eta-squared values (Z^2^) were utilized to assess effect size: small (Z^2^ = 0.02), medium (Z^2^ = 0.13), and large (Z^2^ = 0.26). The MSSE group demonstrated a significant effect size for progress in behavior, mood, and cognition when compared to the activity and control groups. Certainty of evidence: pilot study featuring a limited sample size, suggesting initial evidence with a moderate degree of confidence.	Two-way mixed ANOVAs were employed to examine group variations at different timepoints (pre-, mid-, and posttrial), evaluating the impacts on behavior, mood, cognitive status, and dementia severity.
Hutson et al. 2014 [46]	To evaluate how the Sonas approach impacts individuals with dementia residing in care facilities.	Randomized Controlled Trial.	7–8 weeks.	Size: 39 participants. Age: mean 86.6 years (range 70–99). Gender: 86.1% female.	CSDD, NPI-Q, QoL-AD, Holden Communication Scale, and RAID.	Multisensory stimulation: Sonas intervention with a focus on music and poetry. Senses involved: auditory, tactile, and visual stimuli. Main findings: No notable variances were noted in any of the outcome assessments. The TAU group had a slight advantage in depression scores (CSDD) compared to the standard treatment.	Effect size: No notable differences were observed between the Sonas and treatment-as-usual groups for any of the outcome metrics. The effect size was minor (<0.20), suggesting there was no significant advantage from the intervention. Minimal effect size for the depression measure (CSDD), with r < 0.20. Certainty of evidence: low because of the study’s small sample size and preliminary pilot design.	T-test with independent measures conducted, effect size (r) determined, and no statistically significant results observed.
Sanchez et al. 2016 [47]	To evaluate the impact of MSSE versus personalized music therapy on agitation, mood, anxiety, and cognitive abilities in dementia patients experiencing severe or very severe cognitive deterioration.	Randomized Controlled Trial	16 weeks (32 sessions, two weekly sessions lasting 30 min each).	Size: initial: 22 participants. Final: 18 participants (9 in each group). Age: not specified. Gender: 15 females and 7 males.	CMAI, CSDD, RAID, and SMMSE.	Multisensory stimulation: visual (fiber-optic lights, video, bubble columns), auditory (music), tactile (varied textures), and olfactory (aromatherapy). Senses involved: Visual, tactile, and olfactory. Main findings: MSSE resulted in larger decreases in agitation and anxiety than personalized music. Enhancements in mood were more pronounced in the MSSE group. Cognitive function exhibited slight alterations in both groups, with no notable difference.	Effect size: In terms of agitation, both groups showed improvement in the CMAI total score (F(2,34) = 3.837, *p* = 0.031, η^2^ = 0.166); mood: (F(1,16) = 9.822, *p* = 0.006, η^2^ = 0.374); anxiety: (F(1,16) = 6.500, *p* = 0.021, η^2^ = 0.267); cognitive: No significant effects were reported. Certainty of evidence: moderate given the randomization design.	The extent of the effects is explained qualitatively: MSSE resulted in more significant decreases in agitation and mood issues when compared to music therapy.
Maseda et al. 2014 [48]	To assess the impact of MSSE in a Snoezelen room on the mood and behavior of elderly individuals with dementia in relation to activity-based interventions and control groups.	Randomized Controlled Trial	16 weeks, with 2 weekly sessions lasting 30 min each.	Size: thirty participants separated into three groups of ten. Age: mean age 87.6 years (SD ± 5.7); range 77–96 years. Gender: predominantly female (95%, 19/20 participants).	Behavior and mood: measured using the Interact scale; biomedical parameters: heart rate (beats per minute) and SpO₂ measured before and after sessions.	Multisensory stimulation: visual (fiber optics, water columns, projections), tactile (textured boards, vibrating bed), auditory (music), and olfactory (aroma therapy). Senses involved: visual, tactile, olfactory, and auditory. Main findings: MSSE group: notable enhancements in spontaneous speech (*p* = 0.006), improved social interactions (*p* = 0.014), increased attentiveness (*p* = 0.022), greater enjoyment (*p* = 0.004), and heightened relaxation (*p* = 0.000). Activity group: notable enhancements in happiness (*p* = 0.012), decreased confusion (*p* = 0.043), increased spontaneous speech (*p* = 0.016), social interactions (*p* = 0.005), attentiveness (*p* = 0.002), less boredom (*p* = 0.002), and improved relaxation (*p* = 0.000).	Effect size: high effect sizes: Relating to the environment (Activity: 1.17), relaxed/content (Activity: 1.20), and bored/inactive (Activity: 1.09) show the largest effect sizes, indicating substantial differences. Low effect sizes: Happy/content (MSSE: 0.06), wandering/restless (MSSE: 0.12), and wandering/restless (Activity: 0.14) indicate minimal differences between groups. Certainty of evidence: The research utilized validated instruments (Interact scale) boasting high inter-rater reliability (r = 0.99). Nonetheless, the limited sample size (n = 30) restricts the reliability of the evidence.	Unpaired t-tests: examined variations in mood and behavior among MSSE and activity groups throughout sessions. Paired t-tests: evaluated intra-group variations in behavior and mood prior to and following sessions. Repeated-measures ANOVA: analyzed interactions between groups and time regarding mood, behavior, and biomedical factors. Level of significance: *p* < 0.05 for every test conducted.
Liao et al. 2020 [49]	To assess how garden visits affect the mood, social interactions, cognitive function, and behavioral issues of dementia patients based on staff observations.	Uncontrolled Experimental Study.	Visits to the garden that last approximately 1 h, as indicated by staff.	Size: 42 eligible employees from 9 dementia care centers. Age: between 21–60 years. Gender: all women.	The research utilized a semi-structured questionnaire grounded on a five-point Likert-type scale to evaluate alterations in cognitive traits (e.g., short-term memory, attention, and language skills) and behavioral issues (e.g., aggression, anxiety, and wandering) following visits to the garden. MMSE.	Multisensory stimulation: environmental sensory stimulation (through gardens) Senses involved: all senses. Main findings: The results showed that visits to gardens resulted in enhanced mood, increased social interaction, and advancements in specific cognitive and behavioral areas, along with decreases in behavioral issues like aggression, anxiety, and wandering.	Effect size: The effect sizes for different cognitive and behavioral skills are significant across the categories evaluated. Cognitive skills such as attention (3.60), language skills (3.21), and short-term memory (3.19) demonstrate noticeable advancements, along with behavioral problems like anxiety/agitation (3.69) and mood (3.81). Certainty of evidence: preliminary evidence; restricted because of the small sample size and the observational design.	One-way ANOVA with repeated measures was used to compare mood, social interaction, and cognitive and behavioral traits, while the Mann–Whitney U test assessed differences between groups with differing garden access.
Dixon et al. 2020 [50]	To investigate how people with dementia utilize technology to meet their evolving sensory needs and to pinpoint tactics used by both individuals with dementia and professionals in this specific situation.	Qualitative Study.	There was not a designated treatment duration since the research centered on how individuals with dementia utilized technology in their everyday activities.	Size: There were 30 semi-structured interviews conducted, with 11 interviews involving individuals with dementia and 19 involving practitioners. Age: average age of 61.55 years (SD = 3.503). Gender: All practitioners were women, whereas the individuals with dementia included both men and women.	In-depth understanding of the approaches employed by people with dementia and professionals to adjust to sensory changes, along with the difficulties encountered when utilizing technology.	Multisensory stimulation: sensory changes experienced by individuals with dementia, particularly related to over- or under-stimulation Senses involved: visual and auditory. Main findings: Individuals with dementia frequently faced challenges related to sensory overload during everyday tasks like shopping, reading, and social interactions. Technology was utilized to assist in managing overstimulation (e.g., noise-canceling headphones) and to minimize sensory input, like streamlining visual components on websites. The primary strategies involved inducing at a target level, modifying technology configurations, and relocating devices when changes were inadequate.	Effect size: The research did not determine effect size because it was a qualitative study aimed at descriptive results instead of statistical analysis. Certainty of evidence: The results of the study possess a moderate degree of certainty, as they rely on qualitative data gathered from semi-structured interviews conducted with a small and homogeneous group. There are restrictions concerning the diversity of participants and the ability to generalize.	A constructivist grounded theory method was employed to examine data, organize themes, and confirm them via repeated review. Quantitative statistical analysis was not performed because the study was qualitative in nature.
Cabello et al. 2023 [51]	To evaluate how well a cognitive–multisensory–physical exercise program carried out at home can enhance cognitive and motor skills in individuals with dementia	Uncontrolled Experimental Study.	24 sessions (3 per week) over a period of 8 weeks.	Size: There was a total of 19 participants, with 12 in the experimental group and 7 in the control group. Age: 65–79 years. Gender: 14 males and 5 females.	MMSE, IADL, BI, TUG, gait test, and handgrip strength, dual-task interference (gait and cognitive tasks).	Multisensory stimulation: ROXPro© system. Patients interacted with small devices providing these stimuli, performing motor–cognitive exercises. Senses involved: visual, auditory, and proprioceptive stimuli. Main findings: The experimental group demonstrated notable enhancements in dual-task interference while completing both subtraction and verbal fluency tasks. In the verbal fluency condition, the control group exhibited a notable rise in dual-task interference.	Effect size: η^2^ values were found to be 0.22 for the subtraction task, indicating a moderate effect, and 0.49 for the verbal fluency task, indicating a large effect. Certainty of evidence: high, as the study adhered to ethical guidelines and employed sound statistical methods (ANOVA with repeated measures, post hoc t-tests, and Bonferroni corrections).	The ANOVA findings indicated notable group × time interactions (F = 16.50, *p* < 0.01; η^2^ = 0.49; observed power (OP) = 0.96) for dual-task interference, which reinforces the efficacy of the exercise program.
Alruwaili et al. 2024 [52]	To evaluate how effective a Snoezelen-based multisensory environment, combined with aromatherapy and personal items, is at decreasing agitation in elderly patients with dementia.	Uncontrolled Experimental Study.	Not specified.	Size: 62 participants. Age: 65–90 years. Gender: 53.2% female and 46.8% male.	CMAI, MMSE, and QoL-AD.	Multisensory stimulation: Snoezelen room. Sensor involved: visual, auditory, tactile, and olfactory. Main findings: The treatment effectively decreased restlessness, boosted cognitive abilities, and elevated general health in individuals with dementia. The use of aromatherapy and personal items in a multisensory method had a soothing impact.	Effect size: moderate effect size (f = 0.1) determined through G*Power 3.1.9.2. Certainty of evidence: high, based on the controlled study design and statistical analysis.	Data were examined with a multiple linear regression model at a significance level of α = 0.05 and power of 80%. The research employed intention-to-treat analysis to address dropout rates.
Dolan et al.2022 [53]	To assess the effectiveness of CST and Sonas group treatments for individuals with moderate dementia, specifically targeting cognitive abilities.	Prospective Controlled Study.	Sessions were held twice a week in both inpatient and community settings over a period of time.	Size: 28 participants (25 completed assessments). Age: mean age of 80.29 years (SD = 7.57) Gender: 11 males and 17 females.	NPI, QoL-AD, RBANS, SMMSE, DKEFS, and TT.	Multisensory stimulation: Sonas interventions Sensor involved: auditory, tactile, and visual stimulation. Main findings: The CST group exhibited notable enhancements in cognitive abilities in comparison to the Sonas group, demonstrating a moderate impact on verbal fluency and cognitive function. There were not any notable fluctuations in Sonas.	Effect size: Token Test improvement in the CST group showed a moderate effect size of 0.37. The DKEFS Verbal Fluency also showed a significant difference between groups, with a very small effect size (r = 0.08). Certainty of evidence: moderate; CST showed notable changes within groups, but no differences were seen between groups.	Different types of tests, such as t-test, ANOVA, and Mann–Whitney U test, were utilized, both parametric and non-parametric. The CST group showed statistically significant changes with moderate effect sizes in cognitive improvements.
Bautrant et al. 2022 [54]	To assess how effective various NPTs are at decreasing nighttime BPSDs in older residents living in institutions.	Retrospective Cohort Study.	12 months (January to December 2019).	Size: 84 residents. Age: Mean age 83.6 ± 7.3 years. Gender: 75% female, 25% male.	NPI, MMSE, and AGGIR.	Multisensory stimulation: combination of sensory stimulation. Sensory involved: visual, auditory, tactile, and olfactory. Main findings: Alternative treatments, particularly Montessori-inspired, multisensory activation, and cognitive stimulation, yielded notable progress in decreasing BPSDs. MSSE worked best for wandering, while Montessori-based therapy was most effective for agitation/aggression and screaming. There was a significant decrease in the NPI score from the beginning to the conclusion of the study.	Effect size: Various NPTs showed varying levels of effectiveness, with MSSE proving the best for wandering, while Montessori-based therapy was most effective for agitation/aggression and screaming. Certainty of evidence: high, relying on substantial findings and strong statistical analysis.	The SPSS software (V23) was used to conduct statistical analysis, including the Wilcoxon test, Kruskal–Wallis test, and Mann–Whitney U test. The Bonferroni–Holm correction was employed to account for multiple comparisons. The effect size was considerable, showing *p*-values < 0.0001 in multiple comparisons.
Lorusso et al. 2020 [55]	To investigate how well MSEs work for veterans with dementia, analyzing the views of staff on using MSEs, obstacles to implementing them, and knowledge gained from their use.	Qualitative Study.	From June to August 2018	Size: 32 employees from 12 different locations within the VHA. There was a total of 22 interviews conducted, including 21 individual interviews and 1 group interview with 11 participants. Age: not specified. Gender: not specified.	Employee attitudes towards the efficiency of MSE. Veteran inclinations towards particular MSE components.	Multisensory stimulation: Various elements like bubble tubes, aromatherapy, and music. Sensory involved: visual, auditory, and olfactory. Main findings: MSE had beneficial effects on veterans with dementia, such as decreased agitation, disruptive behavior, and aggression, utilizing calming tools like bubble tubes and aromatherapy.	Effect size: Participants indicated a notable positive effect from MSE, comprising decreased agitation, increased engagement, and enhanced behavior. Elements of MSE like bubble tubes, aromatherapy, and music were frequently mentioned as having positive impacts. Adverse effects: Several participants noted adverse effects, especially for veterans suffering from PTSD, including increased sensitivity to sounds and lights, along with challenges in comprehending MSE stimuli in individuals with severe dementia. Certainty of evidence: grounded in qualitative stories and thematic evaluation.	Rapid Qualitative Inquiry was utilized for coding data and performing thematic analysis. The results illustrate descriptive categories (few, several, and many) determined by the frequency of responses.
Minetou et al. 2023 [56]	To create and build spatial items with integrated technology to improve the quality of life for individuals with dementia through a collaborative design method.	Qualitative Study.	The research was carried out in several phases, with continuous assessment scheduled for the spatial items following their setup.	Size: There were a total of 50 individuals involved, including 30 dementia patients and 10 nurses/caregivers. Age: not specified. Gender: not specified.	Gathering qualitative data through semi-structured interviews, co-design workshops, and sensory ethnography.	Multisensory stimulation: different interventions. Sensory involved: olfactory, tactile, and audio–visual elements. Main findings: Key findings indicate that interactive prototypes (e.g., armchairs and wall frames) may offer sensory experiences including imitated seaside sounds, fragrances, and tactile feelings. These prototypes aim to involve individuals with dementia, aid mobility, and stimulate social connections.	Effect size: not specified. Certainty of evidence: The research employs a qualitative method with continuous iterative assessments. The level of certainty in the evidence is moderate since the results rely on expert evaluations and initial tests that have limited quantitative information.	The study did not include any formal statistical analysis or report effect size. The focus is on qualitative assessment and collaborative design methods to improve prototypes and increase their efficacy in dementia care.

Legend: Mini-Mental State Examination (MMSE), multisensory stimulation (MSSE), activities of daily living (ADLs), Cohen–Mansfield Agitation Inventory (CMAI), Neuropsychiatric Inventory—Nursing Home (NPI-NH), Cornell Scale for Depression in Dementia (CSDD), Severe Mini-Mental State Examination (SMMSE), Bedford Alzheimer Nursing Severity Scale (BANS-S), multisensory environments (MSEs), Veterans Health Administration (VHA), behavioral and psychological symptoms of dementia (BPSDs), Neuropsychiatric Inventory Questionnaire (NPI-Q), Quality of Life–Alzheimer’s Disease Scale (QoL-AD), mild cognitive impairment (MCI), Wechsler Memory Scale-Revises (WMS-R), Consortium to Establish a Registry for Alzheimer’s Disease Animal Naming Test (CERAD-ANT), Boston Naming Test (BNT), Trail-Making-Test B (TMT-B), Beck Depression Inventory (BDI), Magnetic Resonance Imaging (MRI), Threshold, Discrimination, and Identification (TDI), Rating Anxiety in Dementia (RAID), Severe Mini-Mental State Examination (SMMSE), Saturation of Peripheral Oxygen (SpO₂), intensive olfactory training (IOT), Verbal Fluency Test (VFT), Korean Boston Naming Test (K-BNT), Word List Memory Test (WLMT), Word List Recall Test (WLRT), Word List Recognition Test (WLRcT), Constructional Praxis Test (CPT), Constructional Recall Test (CRT), Stroop Color and Word Test (SCWT), Mini-Mental State Examination in Korean version of CERAD (MMSE-KC), Short Geriatric Depression Scale—Korean version (SGDS-K), Lawton Instrumental Activities of Daily Living Scale (IADL), Barthel index (BI), Timed Up and Go (TUG), Cognitive Stimulation Therapy (CST), Neuropsychiatric Inventory (NPI), Repeatable Battery for the Assessment of Neuropsychological Status (RBANS), Delis–Kaplan Executive Function System (DKEFS), Token Test (TT), non-pharmacological therapies (NPTs), Autonomie Gerontologie Groupe Iso Ressources (AGGIR), Post-Traumatic Stress Disorder (PTSD).

**Table 3:** Descriptions and advantages of MSS therapies.

Type of Multisensory Stimulation	Mechanism of Action	Intervention Duration and Number of Sessions	Dementia Conditions for Applications (Based on Findings)	Benefits and Practical Application of the Technique	Specific Observed Results
Snoezelen Multisensory Environments	Uses a controlled sensory environment combining light, sound, and tactile stimuli to enhance mood and reduce agitation.	Typically, 30 min sessions, 2 times per week, over 12–16 weeks.	Severe dementia	Reduces neuropsychiatric symptoms, improves mood, and promotes engagement in institutional settings.	Immediate improvements in mood, behavior, and physiological parameters (heart rate and oxygen saturation). Enhanced visual tracking of stimuli and increased relaxation during sessions. Some studies show a reduction in agitation and improvement in neuropsychiatric symptoms over time, but long-term effectiveness can vary.
Nature-Based Interventions	Leverages natural environments to stimulate senses, improve mood, and foster social interaction.	Unrestricted or scheduled garden or nature environmental visits, varying from daily to weekly interventions.	Mild-to-moderate dementia	Enhances mood, reduces depression, improves attention, and fosters social connections.	Positive influence on mood, social interaction, depression, and agitation. Improvements in attention, time orientation, long-term memory, language, spatial ability, and reduced aggression, especially with unrestricted garden access. Limited access still offers some benefits but to a lesser extent.
Technology-Driven Multisensory Stimulation	Employs digital tools like apps, wearables, and sensory devices to provide customized sensory stimulation and dual-task training.	Varies by device, e.g., 24 sessions of home-based dual-task training in 6–8 weeks.	Mild-to-moderate dementia	Promotes dual-task abilities, improves cognitive engagement, and supports home-based rehabilitation programs.	Improvements in dual-tasking performance (walking while performing a verbal fluency task). Potential for enhancing functional cognition, the ability to apply cognitive skills in daily life. Improvements in verbal fluency during ambulation. Mobile health technology shows promise for delivering combined interventions (cognitive, multisensory, and physical).
Culturally Adapted Multisensory Therapy	Combines sensory activities like Snoezelen and aromatherapy with culturally relevant elements.	Duration and frequency tailored to the cultural context; typically, twice a week for 8–12 weeks.	Applicable across various dementia severities	Reduces agitation, improves neuropsychiatric symptoms, and enhances quality of life in culturally sensitive ways.	Significant reductions in agitation behaviors and improvements in quality of life. Effective for managing nocturnal BPSDs (such as wandering). Cultural adaptation is crucial for maximizing effectiveness. This approach demonstrates the importance of tailoring interventions to individual and cultural backgrounds.
Sonas Program	Group-based sensory therapy incorporating music, tactile activities, and memory games to enhance cognitive and emotional responses	Typically, 14 sessions over 7 weeks, with each session lasting approximately 45 min.	Moderate-to-severe dementia	Enhances cognitive function, improves caregiver quality of life, and fosters communication skills.	Improvements in general cognitive function (MMSE), language skills, and verbal fluency (compared to Sonas itself in comparative studies). Well-tolerated, but no significant benefits on other cognitive measures such as memory, attention, or ADLs were consistently found. This suggests a more targeted effect on specific cognitive domains.

Legend: behavioral and psychological symptoms of dementia (BPSDs), Mini-Mental State Examination (MMSE), activities of daily living (ADL).

## Data Availability

No new data were created or analyzed in this study.
